# Acute-Phase Serum Amyloid A as a Marker of Insulin Resistance in Mice

**DOI:** 10.1155/2008/230837

**Published:** 2008-06-22

**Authors:** Ludger Scheja, Barbara Heese, Heike Zitzer, Mervyn D. Michael, Angela M. Siesky, Heike Pospisil, Ulrike Beisiegel, Klaus Seedorf

**Affiliations:** ^1^Department of Biochemistry and Molecular Biology II, University Medical Center, 20246 Hamburg, Germany; ^2^Former Lilly Research Laboratories, 22419 Hamburg, Germany; ^3^Lilly Research Laboratories, Lilly Corporate Center, Indianapolis, IN 46285, USA; ^4^Center for Bioinformatics, University of Hamburg, 20146 Hamburg, Germany

## Abstract

Acute-phase serum amyloid A (A-SAA) was shown recently to correlate with obesity and insulin resistance in humans. However, the mechanisms linking obesity-associated inflammation and elevated plasma A-SAA to insulin resistance are poorly understood. Using high-fat diet- (HFD-) fed mice, we found that plasma A-SAA was increased early upon HFD feeding and was tightly associated with systemic insulin resistance. Plasma A-SAA elevation was due to induction of *Saa1* and *Saa2* expression in liver but not in adipose tissue. In adipose tissue *Saa3* was the predominant isoform and the earliest inflammatory marker induced, suggesting it is important for initiation of adipose tissue inflammation. To assess the potential impact of A-SAA on adipose tissue insulin resistance, we treated 3T3-L1 adipocytes with recombinant A-SAA. Intriguingly, physiological levels of A-SAA caused alterations in gene expression closely resembling those observed in HFD-fed mice. Proinflammatory genes (*Ccl2, Saa3*) were induced while genes critical for insulin sensitivity (*Irs1, Adipoq, Glut4*) were down-regulated. Our data identify HFD-fed mice as a suitable model to study A-SAA as a biomarker and a novel possible mediator of insulin resistance.

## 1. INTRODUCTION

An important aspect of obesity-linked insulin resistance is chronic, subclinical inflammation taking place in adipose tissue and other major metabolic tissues. Local activation of proinflammatory pathways results in the suppression of insulin signaling thereby contributing to impaired glucose metabolism (see [1] for a recent review). The molecular and cellular mechanisms linking obesity-associated inflammation to insulin resistance are under intense investigation. Within these studies, plasma inflammation markers such as acute-phase proteins [2] draw special attention as they are important both for understanding the relative contribution of inflammation to insulin resistance and their immediate biological activities on insulin signaling as well as for the development of clinical biomarkers.

One acute-phase protein reported to be increased in plasma of obese and insulin resistant humans is serum amyloid A (SAA) (reviewed in [3]). Acute-phase SAA (A-SAA) consists of two closely related isoforms, SAA1 and SAA2, which can be induced over a wide expression range in response to proinflammatory stimuli. Additional members of the SAA family are SAA3 and SAA4 [3]. SAA3 is expressed in mice and other mammalian species but apparently not in humans who have a defective *Saa3* gene [4]. SAA3 is also induced under proinflammatory conditions and may share physiological functions with the A-SAA isoforms. SAA4 is a constitutively expressed member of the family and responds only moderately to inflammatory stimuli.

Chronic systemic elevation of A-SAA has been linked to metabolic disease and is a well-established risk factor of atherosclerosis [5–7]. It also appears to correlate with insulin resistance and body mass index in humans, as demonstrated in recent studies. A-SAA levels in patients fell when either fat mass was reduced or insulin sensitivity was restored by treatment with a PPAR*γ* agonist [8–10]. Interestingly, although liver is the organ believed to be most important for A-SAA secretion in the acute-phase response, in insulin resistance-related human studies *Saa1* and *Saa2* expression was higher in adipose tissue than in liver [9, 11, 12] and also the correlation of A-SAA gene expression with plasma levels was higher in this tissue [11]. Thus, it appears that the regulation of A-SAA in the context of obesity and insulin resistance has some unique features as compared to the acute-phase response. A-SAA induction in that context is triggered primarily by cytokines such as interleukin (IL)-1 and tumor necrosis factor-*a* (TNF *a*), but also by IL-6 and related cytokines [2, 13].

In addition to their biomarker properties, SAA isoforms may also be causally involved in the development of metabolic diseases. In the case of atherosclerosis, a couple of mechanisms have been proposed. A-SAA is an apolipoprotein predominantly associated with high-density lipoprotein (HDL) particles and, when increased, it can influence antiatherogenic functions of HDL, especially such involving cholesterol uptake and efflux [14–16]. Also, as shown in mice, A-SAA binds to atherosclerotic lesions and, thereby, may cause retention of lipoproteins and lipid deposition [17]. Biological functions of A-SAA potentially contributing to both atherosclerosis and insulin resistance are proinflammatory properties normally involved in pathogen defense and tissue repair such as chemotaxis, cytokine induction, and the secretion of extracellular matrix-degrading proteases [18–20]. Chronic activation of these inflammatory processes may lead to infiltration of immune cells into the tissues. In obesity, massive infiltration of adipose tissue by macrophages takes place which can be detected through increased expression of myeloid cell markers such as F4/80, CD68, and CD11b [21, 22]. This infiltration is believed to be triggered by increased secretion by adipose tissue cells of chemoattractant molecules including the chemokine CCL2 (also known as MCP1) [23]. Accumulation of activated macrophages leads to sustained production of proinflammatory mediators, entailing adipocyte insulin resistance, and deteriorated glucose metabolism.

Studies relating to A-SAA induction in rodents have been performed in mouse atherosclerosis models and have offered valuable clues regarding A-SAA regulation by lipids [24–26]. However, they were not designed to study defects or mechanisms relating to glucose homeostasis. In order to address the role of SAA in glucose metabolism, we designed a study using a common model of insulin resistance, the diet-induced obesity (DIO) mouse. We induced distinct degrees of overweight and insulin resistance by feeding a high-fat diet (HFD) for different periods of time and compared plasma A-SAA levels and expression of SAA isoforms with parameters of insulin sensitivity and inflammation in liver, adipose tissue and muscle. Furthermore, using cultured adipocytes we investigated whether A-SAA may directly promote the expression of proinflammatory genes and interfere with insulin signaling.

## 2. MATERIALS AND METHODS

### 2.1. Materials

Recombinant A-SAA was obtained from Peprotech (Hamburg, Germany). DMEM, BSA, and calf serum were from Invitrogen (Karlsruhe, Germany). FCS was from Biochrom-Seromed (Berlin, Germany). All other reagents (mouse TNF*α*, insulin, dexamethasone, IBMX, LPS, PMB) were purchased from Sigma-Aldrich (Munich, Germany).

### 2.2. Animal diet regimens and necropsy

Age-matched male C57Bl/6 mice (Taconic, Germantown, NY) on distinct diets were used as models of insulin resistance and as the respective controls. Severe insulin resistance and obesity were induced by weaning animals on an HFD (Bioserv3282, 59 cal% fat) and maintaining them on the diet for 16 weeks. Mildly insulin-resistant mice were generated by randomizing lean mice based on fasting plasma glucose and insulin, and subsequently feeding one group the HFD for one week while the other mice were kept as controls on a standard rodent chow (Purina5001).

For randomization, animals were bled by tail clip method at 19 weeks of age after overnight fast. At 20 weeks of age, all mice were subjected to necropsy. The animals were fasted overnight (16 hours), anesthetized with isoflurane, and cardiac stick was performed to collect blood as EDTA plasma from the right ventricle of the heart. After bleeding, the animals were perfused (10 U/L heparinized saline) through the right ventricle of the heart, allowing the blood to exit the animal through a cut in the inferior vena cava. Organs were then removed. Selected organs and blood were snap frozen in liquid nitrogen and stored at −80°C.

Animals were maintained on a 12-hour light, 12-hour dark cycle and received house water ad libitum. All animals were kept in accordance with the Institutional American Association for the Accreditation of Laboratory Animal Care guidelines.

### 2.3. Plasma parameters

Plasma insulin was determined using Rat/Mouse Insulin Assay kit (Mesoscale Discovery, Gaithersburg, Md) and Mouse Endocrine Lincoplex kit (Millipore, St. Charles, Mo) for randomization and final study, respectively. Glucose was measured using a Hitachi 912 clinical chemistry analyzer (Roche, Indianapolis, Ind). Adiponectin and A-SAA were determined using ELISAs from R&D Systems (Minneapolis, Minn) and Biosource (Invitrogen), respectively.

### 2.4. Total RNA extraction and real-time quantitative PCR

Total RNA from frozen tissue samples was extracted using the TissueLyser and RNeasy system (Qiagen, Hilden, Germany) according to the manufacturer's instructions (RNeasy Lipid Tissue Kit and RNeasy Fibrous Tissue Kit for adipose and muscle tissue, resp.). DNA contaminants were removed via column DNase treatment (RNase-Free DNase set; Qiagen). 1 *μ*g of RNA was reversely transcribed in a 50 *μ*L reaction mixture using the High Capacity cDNA Archive Kit (Applied Biosystems, Darmstadt, Germany) according to the manufacturer's instructions. Real time RT-PCR was performed as described previously [27]. For detections of *Saa2* and *Irs2*, Assay-by-Design Assay that mixes with the following sequences was used:

Irs2-fwd GCGGCCTCATCTTCTTCACT,

mIrs2-rev AACTGAAGTCCAGGTTCATATAGTCAGA,

mIrs2-Pr CGACAGCCGGCAGCGCTCTC,

mSaa2-fwd GAGTCTGCCATGGAGGGTTTT,

mSaa2-rev TGTAGGCTCGCCACATGTC,

mSaa2-Pr TCCAGCCCCTTGGAAAG.

For all other genes Assay-on-Demand primer/probe sets supplied by Applied Biosystems were used (Assay IDs are available upon request). Relative expression was calculated by normalization to selected house keeper mRNA (cyclophilin E for muscle, TBP for all others) by ΔΔCt method [28]. Data are reported as copy number relative to 10^4^ copies of house keeper.

### 2.5. Differentiation and treatment of 3T3-L1 adipocytes

3T3-L1 cells were maintained in DMEM containing 10% calf serum. To differentiate, cells were grown to confluence, switched to differentiation medium (DMEM, 4.5 g/L glucose, supplemented with 10% FCS, 5 *μ*g/mL insulin, 0.25 *μ*M dexamethasone, 0.5 mM IBMX), and cultured for 2 days. Subsequently, cells were kept in differentiation medium without dexamethasone and IBMX for 3 days and further differentiated in medium also lacking insulin for at least 1 week before experimentation. Before treatment, cells were washed once with PBS and starved overnight in DMEM, 1 g/L glucose, containing 0.1% BSA. Reagents were then added directly to wells.

### 2.6. Statistical analysis

Unless indicated otherwise two-tailed Student's *t*-test was used to assess statistical significance between groups. Pearson's correlation test was used to evaluate the degree and the significance of association between plasma A-SAA and obesity or insulin resistance parameters.

## 3. RESULTS

### 3.1. High-fat diet feeding induces insulin resistance and increases plasma A-SAA levels

**Figure 1:**
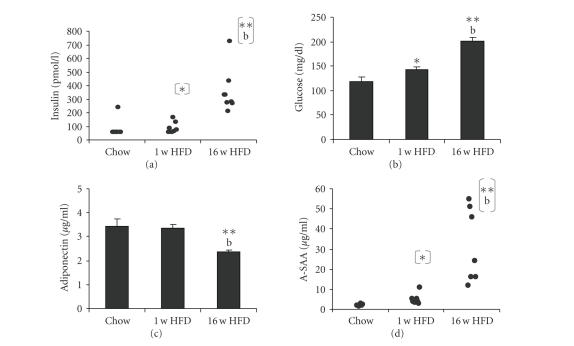
(a) Fasting plasma insulin, (b) glucose, (c) adiponectin, and (d) A-SAA levels in mice fed either control diet (Chow), control diet for 15 weeks, and HFD for one week (1 w HFD), and HFD for 16 weeks (16 w HFD), respectively. Bar graphs are presented as mean ±SEM (*n* = 5–8), statistics:*P < .05 versus Chow, **P < .01 versus Chow, ^(a)^ *P* < .05 versus 1 w HFD, ^(b)^ *P* < .01 versus 1 w HFD; Mann-Whitney U-Test was used for insulin (7/8 measurements in Chow group were below detection level, 56 pmol/L); two-tailed Student's *t*-test for all other measurements.

One week HFD (1 w HFD) feeding of mice resulted in enhanced body weight (27.0 ± 0.7 g versus 23.0 ± 0.5 g, mean ± SEM, *P* < .001) and induced significant fasting hyperinsulinemia and hyperglycemia compared to controls maintained on chow diet (see Figure 1), indicating mild insulin resistance already after a short period on the obesigenic diet. As expected, 16 weeks of HFD (16 w HFD) feeding led to more pronounced overweight (44.9 ± 0.7 g; *P* < .001 versus controls) and insulin resistance compared to controls (see Figure 1). Plasma leptin showed a similar pattern (19 ± 5 pmol/L, control, 130 ± 25 pmol/L, 1 w HFD, 1205 ± 194 pmol/L, 16 w HFD; *P* < .001).

Plasma A-SAA was elevated in the 1 w HFD group (see Figure 1; 5.06 ± 1.05 *μ*g/mL versus 2.11 ± 0.25 *μ*g/mL in control animals, *P* < 0.05) and increased substantially further in the 16 w HFD group (31.4 ± 7.0 *μ*g/mL, *P* < .01 versus controls), demonstrating that the plasma levels of A-SAA are associated with obesity and insulin resistance in a quantitative fashion. This quantitative association was confirmed by combining all experimental groups and performing a correlation analysis for plasma A-SAA (Pearson correlation coefficients: 0.76, insulin; 0.75, glucose; 0.84, HOMA-IR; 0.78, leptin; *P* < .001). In contrast, plasma adiponectin levels were not affected by 1 w HFD, however, they were significantly reduced after 16 w HFD (see Figure 1).

### 3.2. Tissue-specific induction of SAA isoforms

**Figure 2:**
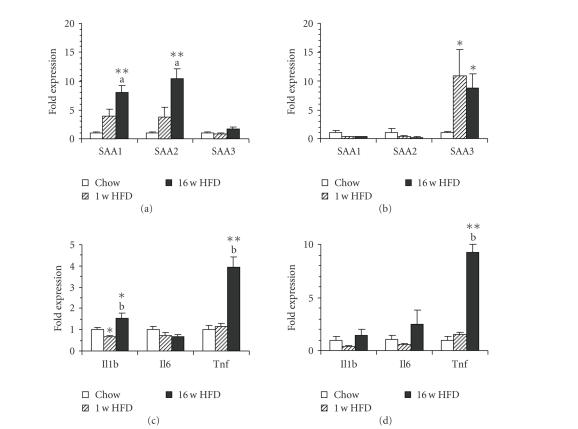
(a), (b) Diet-induced expression of SAA isoforms and (c), (d) SAA inducers in (a), (c) liver and (b), (d) adipose tissue, quantified by real-time quantitative PCR. Data are mean ±SEM normalized to control animals (fold over Chow). Number of animals (*n*), see Table 1. Statistics:*P < .05 versus Chow, **P < .01 versus Chow, ^(a)^ *P* < .05 versus 1 w HFD, ^(b)^ *P* < .01 versus 1 w HFD; two-tailed Student's *t*-test.

In order to understand the contribution of key metabolic tissues to the observed A-SAA induction in plasma, we performed real-time quantitative PCR experiments in liver, adipose tissue, and skeletal muscle. As shown in Figure 2 and Table 1, both A-SAA isoforms, *Saa1* and *Saa2*, were highly expressed in liver and were induced by short- and long-term HFD feeding (*P* < .15 for 1 w HFD). *Saa3* was also well expressed in liver, however, it was not significantly induced in this tissue by the HFD. By contrast, *Saa3* was strongly expressed in adipose tissue and markedly induced already after one week of HFD with no further increase observed after 16 weeks (see Figure 2). Interestingly, expression of *Saa1* and *Saa2* was very low in adipose tissue (see Table 1) and neither of them was induced by the HFD indicating that adipose tissue is not involved in the elevation of plasma A-SAA by the HFD. In skeletal muscle basal expression of all SAA isoforms was very low. While the mRNA expression of *Saa1* and *Saa2* was not affected by HFD, *Saa3* was markedly induced after 16 weeks of HFD (see Table 1).

The cytokines IL-1*β*, IL-6, and TNF*α* are established as important inducers of *Saa1* and *Saa2* in liver during the acute-phase response. In order to assess the role of these cytokines in *Saa1* and *Saa2* induction upon HFD feeding, we quantified also their expression. In liver, the mRNAs of both IL-1*β* and TNF*α* (*Il1b*, *Tnf*) were increased after 16 w HFD feeding. In contrast, neither cytokine was induced after 1 w HFD feeding, suggesting that they are not responsible for the early elevation of plasma A-SAA (see Figure 2).

In adipose tissue, *Tnf* was strongly induced after 16 weeks of HFD, however, only a trend toward an increase was observed in the 1 w HFD group. The expression level of *Il1b* in adipose tissue was not significantly changed by HFD feeding (see Figure 2).

The mRNA of IL-6 (*Il6*) was not induced in any of the tissues examined in a statistically significant manner (see Figure 2 and Table 1).

### 3.3. Association of A-SAA levels with induction of macrophage markers and chemoattractant molecules in insulin target tissues

**Figure 3:**
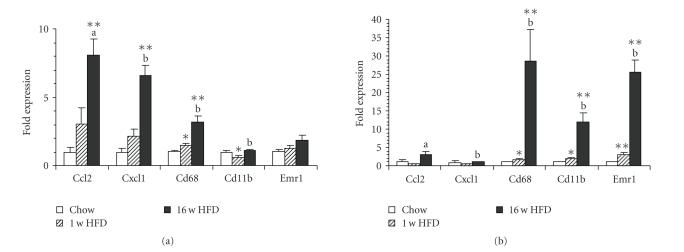
(a) Diet-induced expression of inflammatory genes in liver and (b) adipose tissue, quantified by real-time quantitative PCR. Data are mean ±SEM normalized to control animals (fold over Chow). Number of animals (*n*), see Table 2. Statistics:*P < .05 versus Chow, **P < .01 versus Chow, ^(a)^ *P* < .05 versus 1 w HFD, ^(b)^ *P* < .01 versus 1 w HFD, two-tailed Student's *t*-test.

The observed tissue- and isoform-selective patterns of SAA expression in response to the high-fat diet prompted us to further characterize the underlying inflammatory processes. To this end, we assessed the expression of inflammatory markers including the chemoattractant molecules CCL2 and CXCL1 (also known as KC) as well as macrophage infiltration markers (CD68, CD11b, F4/80). As shown in Figure 3 and Table 2, one week feeding of HFD slightly induced the expression of the chemokine genes (*Ccl2, Cxcl1*) in liver (*P* < .15) while neither chemokine was increased in adipose tissue and only *Ccl2* was induced in muscle. The genes of the myeloid cell markers CD68, CD11b, and F4/80 (*Emr1*) were moderately but significantly induced in adipose tissue after one week of HFD, demonstrating incipient adipose infiltration by macrophages, while in the other tissues significant induction of these markers could not be observed, except for *Cd68* in liver.

After 16 weeks of HFD, pronounced expression of inflammatory markers was observed in all tissues examined (see Figure 3 and Table 2). The macrophage markers *Cd68*, *Cd11b*, and *Emr1* were highly induced in adipose, moderately increased in muscle, while in liver only *Cd68* was induced significantly.

Taken together, the temporal expression pattern of liver *Ccl2*, *Cxcl1*, and *Cd68* as well as adipose tissue *Cd68*, *Cd11b*, and *Emr1* corresponded to liver *Saa1* and *Saa2* mRNA, plasma A-SAA protein, and insulin resistance in our experiment. Interestingly, none of the inflammatory markers in adipose tissue showed an expression pattern similar to *Saa3*.

### 3.4. Reduced expression of genes implicated in insulin sensitivity in liver, adipose tissue, and skeletal muscle of HFD-fed mice

**Figure 4:**
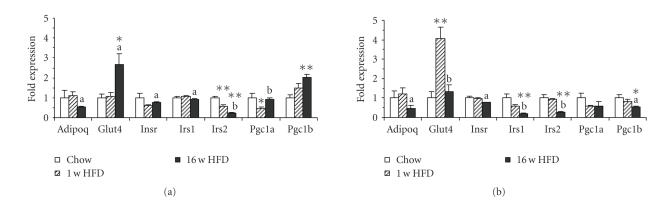
(a) Diet-induced expression of genes related to insulin sensitivity in liver and (b) adipose tissue, quantified by real-time quantitative PCR. Data are mean ±SEM normalized to control animals (fold over Chow). Number of animals (*n*), see Table 2. Statistics:*P < .05 versus Chow, **P < .01 versus Chow, ^(a)^ *P* < 0.05 versus 1 w HFD, ^(b)^ *P* < .01 versus 1 w HFD, two-tailed Student's *t*-test.

Next, we determined the effect of HFD feeding on the expression of signaling molecules known to regulate insulin sensitivity (see Figure 4 and Table 2). Consistent with unaltered plasma adiponectin levels after one week and reduced plasma adiponectin levels after 16 w HFD feeding, the mRNA levels of the adiponectin gene (*Adipoq*) in adipose tissue were not changed after one week and significantly reduced after 16 w HFD feeding (see Figure 4). *Adipoq* levels in liver showed a similar temporal pattern upon HFD feeding, however expression was very low compared to adipose tissue (see Table 2). Expression of the insulin-responsive glucose transporter *Glut4* (also known as Slc2a4) was induced 6-fold after 1 w HFD in adipose tissue and returned to basal levels after 16 w HFD while in muscle it was moderately but significantly suppressed already after 1 w HFD (see Table 2).

The expression of insulin receptor substrate-1 (*Irs1*) was largely unaffected in liver and skeletal muscle while it was progressively down-regulated in adipose tissue. By contrast, *Irs2* expression was reduced in all three tissues in a tissue-specific manner (see Figure 4 and Table 2).

To examine potential HFD-mediated tissue specific shifts in metabolic capacity, we determined the expression of PGC-1*α* and PGC-1*β*, two transcriptional regulators of mitochondriogenesis and oxidative metabolism. As shown in Figure 4 and Table 2, *Pgc1a* expression was significantly reduced after 1 w HFD in liver and recovered to control levels after 16 w HFD. In adipose tissue and skeletal muscle, only a trend towards downregulation could be observed. The expression of *Pgc1b* was upregulated in liver and skeletal muscle after 16 w HFD with a trend towards upregulation evident already after 1 w HFD in liver. In adipose tissue, *Pgc1b* was reduced after 1 w HFD diet and expression was further impaired after 16 w HFD.

In summary, significant tissue-specific downregulation of genes critical for maintaining insulin sensitivity was observed in the HFD-fed mice.

### 3.5. Effects of recombinant A-SAA on gene expression and metabolism in 3T3-L1 adipocytes

**Figure 5:**
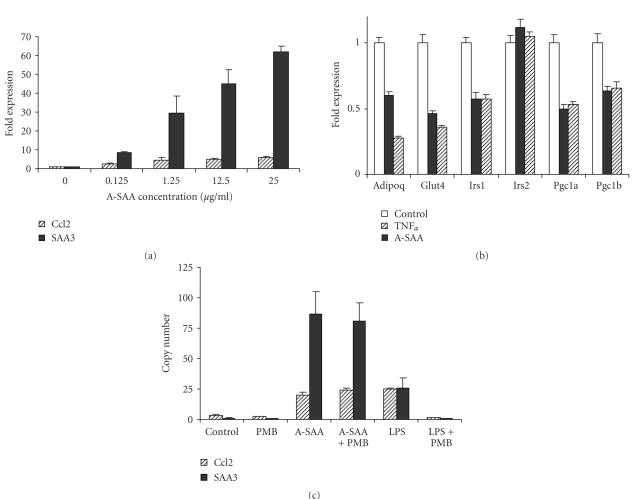
Effects of recombinant A-SAA and TNF*α* on gene expression in differentiated 3T3-L1 adipocytes. (a) Dose-dependent induction of *Saa3* and *Ccl2* by recombinant A-SAA. Differentiated 3T3-L1 adipocytes were washed with PBS and starved overnight in DMEM containing 0.1% BSA. Subsequently, cells were treated for 24 hours with various concentrations of recombinant A-SAA (0, 0.125 1.25, 12.5, 25 *μ*g/mL). (b) Regulation of signaling genes by A-SAA and TNF*α*: Differentiated 3T3-L1 adipocytes were treated as described under (a) and subsequently incubated with A-SAA (12.5 *μ*g/mL, closed bars) and TNF*α* (10 ng/mL, hatched bars) for 24 hours. (c) Effect of polymyxin B (PMB) on induction of *Saa3* and *Ccl2*. PMB at 10 *μ*g/mL was added alone or in combination with lipopolysaccharide (LPS, 2.5 ng/mL) and A-SAA (1.25 *μ*g/mL), respectively. Experimental conditions were as described under (a). Copy numbers are relative to one copy of TBP mRNA. Representative data from at least three independent experiments are shown. Data are mean ± standard deviation relative to untreated controls.

The observed concurrence of elevated plasma A-SAA with hyperinsulinemia in this mouse model prompted us to study a potential causal role of A-SAA in the development of peripheral insulin resistance, using differentiated 3T3-L1 cells as a model system. Treatment with physiological concentrations of recombinant A-SAA for 24 hours led to a pronounced induction of *Ccl2* and *Saa3* in a dose-dependent manner (see Figure 5).

These effects were unlikely to be caused by endotoxin contamination of the A-SAA preparation, as they were unaffected by the addition of the endotoxin neutralizer polymyxin B (see Figure 5 and [29]). In contrast, genes involved in insulin signaling, glucose transport, and mitochondriogenesis (*Adipoq*, *Irs1*, *Glut4*, *Pgc1a*, *Pgc1b*) were significantly downregulated by A-SAA in a degree similar to TNF*α* treatment (see Figure 5). Interestingly, the alterations in 3T3-L1 gene expression resembled but were not identical to those observed in vivo in adipose tissue after chronic HFD feeding (see Figure 4). Specifically, *Irs2* was downregulated in vivo but not in vitro, while *Glut4* was downregulated in vitro but not in vivo.

Next, we checked whether the observed changes in gene expression had effects on glucose transport, an important insulin-regulated metabolic function of adipocytes. Treatment of 3T3-L1 adipocytes with A-SAA for 24 hours had no significant effect on insulin-stimulated glucose transport either at submaximal or maximum insulin concentrations (data not shown).

## 4. DISCUSSION

We report here the effect of HFD feeding on SAA in the insulin resistance- and obesity-prone mouse strain C57Bl/6. Short-term HFD feeding (1 w HFD) resulted in a moderate but significant increase in fasting plasma insulin, glucose and leptin, indicating incipient, and mild insulin resistance in this group. The chronically fed group (16 w HFD) was severely insulin resistant, as shown by elevated fasting plasma insulin, glucose and leptin, and reduced adiponectin levels. In order to relate the observed insulin resistance to inflammatory processes, we measured plasma levels of A-SAA and found that these were already elevated after one week of HFD, further increasing after 16 weeks of HFD feeding. A correlation of plasma A-SAA with insulin resistance and obesity in humans has been reported by several groups, however, acute phase proteins as markers or mediators of insulin resistance have not been addressed in animal models so far. We show for the first time that plasma A-SAA is associated with obesity and insulin resistance in the frequently used DIO mouse model. Therefore, this model may be useful to unravel molecular mechanisms relating to A-SAA and its potential significance to insulin resistance.

In order to determine the contribution of metabolically relevant tissues to the diet-induced rise in plasma A-SAA, we quantified the expression of SAA genes. The expression of the genes corresponding to A-SAA, *Saa1*, and *Saa2*, in liver correlated with plasma A-SAA, while in adipose tissue and muscle expression of the A-SAA isoforms was very low and no induction by HFD was observed. This is in contrast to recently published human studies which reported that the elevation of plasma A-SAA in metabolic disease is linked to induction of *Saa1* and *Saa2* in adipose tissue [9, 12] but apparently not in liver [11]. In adipose tissue of the HFD-fed mice, the *Saa3* gene was highly induced suggesting that in mouse adipose tissue SAA3 plays a role similar to adipose A-SAA in humans who do not have a functional *Saa3* gene [4]. Further experiments with regard to *Saa3* gene regulation and functions in murine adipose tissue are needed to understand how closely murine adipose *Saa3* relates to *Saa1* and *Saa2* in human adipose tissue.

The tissue specific and temporal pattern of SAA expression prompted us to further delineate the inflammatory responses in the tissues of the insulin resistant mice by assessing the expression of inflammatory mediators as well as expression of myeloid cell surface markers as indicators of macrophage activation and infiltration. These were then put into relation to plasma A-SAA levels and to the observed SAA expression patterns. In agreement with published data [1, 21, 22], fully insulin resistant mice (16 w HFD) showed a substantial induction in adipose tissue of the cytokine *Tnf*, the chemokine *Ccl2* as well as the macrophage markers *Cd68*, *Cd11b,* and *Emr1*. In liver and in muscle, *Tnf* and *Ccl2* expressions were also induced, however, less pronounced than in adipose tissue while the macrophage markers were only partially and moderately induced in these tissues. One exception was *Cd68* which, like the chemokine *Cxcl1*, showed an expression pattern similar to *Saa1* and *Saa2* in liver suggesting that they may be regulated in a similar manner as the A-SAA isoforms. After one week of HFD feeding, moderate but significant induction of all three macrophage markers was observed in adipose tissue, but not in the other tissues, indicating that adipose macrophage infiltration [21, 22] is an early event related to HFD-induced insulin resistance. Expression of the chemokine CCL2, which has been implicated in obesity-linked macrophage infiltration [23], was, however, not elevated in fat at this stage. These data suggest that other chemoattractants may play an important role in the early phase of adipose macrophage infiltration, while CCL2 may be more important at later stages of obesity-induced adipose tissue inflammation. SAA3 which was profoundly induced in the 1 w HFD group and which has very recently been shown to exert chemotactic activity on monocytes [30] may actually be such a chemoattractant conferring initiation of macrophage infiltration in adipose tissue. Expression of *Saa3* in adipocytes is sensitive to inflammatory stimuli as well as nutrient overflow [31], making it a good candidate for the induction of macrophage infiltration in adipose tissue. Studies involving loss of function of SAA3 or the not yet identified SAA3 receptor responsible for macrophage attraction are needed to definitely address the role of SAA3 in insulin resistant adipose tissue.

In light of the observations that adipose tissue exhibited the first detectable inflammatory response under HFD feeding, and that plasma A-SAA correlates with the degree of insulin resistance, we asked whether there may be a direct induction of insulin resistance in adipocytes by A-SAA. For this purpose, we incubated 3T3-L1 adipocytes with recombinant A-SAA and used the expression of inflammatory markers and signaling genes as indicators for insulin resistance. Intriguingly, physiological A-SAA concentrations led to an induction of *Saa3* and downregulation of selected signaling genes linked to insulin sensitivity such as *Adipoq*, *Glut4*, *Irs1*, *Pgc1a*, and *Pgc1b*. Reduction of these genes, with the exception of *Glut4* and *Pgc1a*, was also detected in adipose tissue of the insulin resistant mice after 16 weeks on the HFD. However, insulin-stimulated glucose transport was not altered after A-SAA treatment in our experiments suggesting that A-SAA alone is not sufficient to induce impaired insulin signaling in adipocytes. Interestingly, in contrast to adipose tissue after chronic HFD feeding, the expression of *Irs2* in the 3T3-L1 adipocytes was unaltered which is likely explaining the preserved insulin sensitivity. Nevertheless, it is conceivable that A-SAA secreted from the liver potentiates inflammatory and insulin desensitization processes in adipose tissue. This mechanism would likely be more important in an early phase of insulin resistance, comparable to our 1 w HFD group, when tissue expression of inflammatory regulators such as TNF*α*, which is well known to have the effects described above on adipocytes [32, 33] and mediates insulin resistance in vivo [1], is not yet significantly induced.

What could be the primary factor causing A-SAA induction in liver? Known inducers of the A-SAA isoforms in this tissue are the cytokines IL-1*β*, IL-6, and TNF*α* [3, 13]. Only the latter was induced to a meaningful degree in liver in our study, with a strong rise after 16 weeks on the HFD, however, no change compared to the controls after one week on the HFD. Since we found no significant change in expression of these cytokines in the other tissues investigated, it is unlikely that either of these cytokines plays a role in the early rise of *Saa1* and *Saa2* in liver, and hence the elevation of plasma A-SAA.

An alternative mechanism of SAA elevation in liver is lipid overload. Evidence for a role of lipids in A-SAA induction was provided in dietary studies with mouse atherosclerosis models showing an induction of *Saa1*, *Saa2*, and *Saa3* in liver by atherogenic diets containing high fat and high cholesterol [24, 25, 34]. Similarly, in humans plasma A-SAA could be induced by a high-cholesterol diet [35]. Since the diet used in our experiment also contained cholesterol, albeit less than in a typical atherogenic diet, it is possible that cholesterol played an important role here as well. However, in our study total liver cholesterol and triglycerides were not elevated in the 1 w HFD group (data not shown), arguing against a simple lipid overload mechanism. Clearly, more studies are warranted to unravel the nutritional mechanism of SAA induction in humans and animal models in order to understand its respective contributions to atherosclerosis and impaired glucose metabolism as well as its value as a clinical biomarker.

Taken together, we show that in the commonly used DIO mouse model plasma A-SAA levels are associated with insulin resistance. Our data indicate that A-SAA elevation is due to *Saa1* and *Saa2* induction in liver but not, as reported for humans, in adipose tissue. Also, we identify *Saa3* as a strong candidate for mediating the initiation of adipose tissue inflammation in HFD-induced obesity. Furthermore, we found that recombinant A-SAA at physiological concentrations regulates gene expression in cultured adipocytes in a fashion similar to insulin resistant adipose tissue, suggesting that A-SAA might be a contributor to the development of insulin resistance and not merely a marker of inflammation. Future experiments will be directed towards the triggering mechanisms for SAA induction in liver and adipose tissue, and the importance of adipose tissue derived SAA3 in provoking inflammatory responses and insulin resistance.

## Figures and Tables

**Table 1:** Expression of SAA isoforms and SAA inducers in liver, adipose tissue and skeletal muscle of HFD-fed mice. Data are reported as copy numbers relative to 10^4^ copies of house keeper. Numbers of animals (*n*) are in parentheses. Data are mean ±SEM;*P < .05 versus Chow, **P < .01 versus Chow, ^(a)^ *P* < .05 versus 1 w HFD, ^(b)^ *P* < .01 versus 1 w HFD; two-tailed Student's *t*-test.

Experimental Groups	Chow	1 w HFD	16 w HFD
	Copy number	Copy number	Copy number
Liver			
*Saa1*	20,885 ± 3,746 (5)	80,450 ± 27,367 (6)	168,070 ± 24,441 (5)**^(a)^
*Saa2*	96,460 ± 16,927 (5)	370,729 ± 150,376 (6)	998,406 ± 164,697 (5)**^(a)^
*Saa3*	18,824 ± 4,753 (6)	16,098 ± 3,374 (6)	31,641 ± 7,118 (6)
*Il1b*	1,216 ± 138 (6)	813 ± 87 (6)*	1,885 ± 255 (6)*^(b)^
*Il6*	30 ± 5 (6)	22 ± 4 (6)	21 ± 3 (6)
*Tnf*	296 ± 65 (6)	339 ± 51 (6)	1,159 ± 142 (6)**^(b)^
Adipose tissue			
*Saa1*	262 ± 86 (6)	70 ± 21 (6)	78 ± 17 (6)
*Saa2*	1,544 ± 1,035 (5)	615 ± 170 (4)	355 ± 75 (5)
*Saa3*	5,566 ± 1,086 (5)	60,924 ± 25,363 (4)*	48,600 ± 14,279 (5)*
*Il1b*	261 ± 88 (5)	97 ± 21 (4)	384 ± 142 (5)
*Il6*	253 ± 105 (6)	139 ± 24 (6)	618 ± 352 (6)
*Tnf*	648 ± 189 (6)	1,015 ± 119 (6)	6,009 ± 488 (6)**^(b)^
Skeletal muscle			
*Saa1*	24 ± 4 (6)	23 ± 6 (6)	36 ± 4 (6)
*Saa2*	92 ± 18 (6)	75 ± 12 (6)	96 ± 16 (6)
*Saa3*	44 ± 21 (6)	65 ± 23 (6)	1,196 ± 210 (6)**^(b)^
*Il1b*	34 ± 6 (6)	41 ± 8 (6)	45 ± 5 (6)
*Il6*	88 ± 10 (6)	96 ± 13 (6)	56 ± 5 (6)*^(a)^
*Tnf*	60 ± 20 (6)	115 ± 30 (6)	122 ± 7 (6)*

**Table 2:** Expression of inflammatory genes and genes related to insulin sensitivity in liver, adipose tissue and skeletal muscle of HFD-fed mice. Data are reported as copy number relative to 10^4^ copies of house keeper. Numbers of animals (*n*) are in parentheses. Data are mean ±SEM;*P < .05 versus Chow, **P < .01 versus Chow, ^(a)^ *P* < .05 versus 1 w HFD, ^(b)^ *P* < .01 versus 1 w HFD; two-tailed Student's *t*-test.

Experimental Groups	Chow	1 w HFD	16 w HFD
	Copy number	Copy number	Copy number
Liver			
*Ccl2*	590 ± 179 (6)	1,813 ± 692 (6)	4,769 ± 677 (6)**^(a)^
*Cxcl1*	2,074 ± 529 (6)	4,465 ± 1,016 (6)	13,709 ± 1,489 (6)**^(b)^
*Cd68*	8,969 ± 880 (6)	13,192 ± 1,621 (6)*	28,417 ± 3,828 (6)**^(b)^
*Cd11b*	2,129 ± 223 (6)	1,300 ± 278 (6)*	2,317 ± 156 (6)^(b)^
*Emr1*	2,789 ± 588 (6)	3,525 ± 646 (6)	5,186 ± 977 (6)
*Adipoq*	61 ± 23 (6)	67 ± 11 (6)	33 ± 3 (6)^(a)^
*Glut4*	80 ± 15 (6)	84 ± 15 (6)	212 ± 44 (6)*^(a)^
*Insr*	9,416 ± 1,909 (4)	5,795 ± 223 (4)	7,275 ± 380 (4)^(a)^
*Irs1*	19,376 ± 1,196 (6)	20,830 ± 905 (6)	18,087 ± 355 (6)^(a)^
*Irs2*	55,531 ± 4,549 (6)	31,257 ± 4,180 (6)**	13,435 ± 832 (6)**^(b)^
*Pgc1a*	7,065 ± 1,584 (6)	3,357 ± 460 (6)*	6,348 ± 559 (6) ^(b)^
*Pgc1b*	6,257 ± 1,012 (6)	9,217 ± 1,576 (6)	12,543 ± 1,083 (6)**
Adipose tissue			
*Ccl2*	10,700 ± 6,564 (5)	6,628 ± 628 (5)	32,055 ± 8,522 (6)^(a)^
*Cxcl1*	1,861 ± 752 (6)	802 ± 129 (6)	1,859 ± 284 (6)^(b)^
*Cd68*	51,947 ± 5,446 (6)	92,213 ± 15,472 (6)*	1,492,178 ± 435,981(6)**^(b)^
*Cd11b*	19,011 ± 3,357 (6)	35,227 ± 4,969 (6)*	227,310 ± 49,105 (6)**^(b)^
*Emr1*	2,187 ± 381 (6)	6,618 ± 1,032 (6)**	55,752 ± 7,298 (6)**^(b)^
*Adipoq*	1,658,946 ± 598,784 (6)	2,028,698 ± 485,799 (6)	803,801 ± 248,995 (6)^(a)^
*Glut4*	19,418 ± 6,662 (6)	78,678 ± 11,679 (6)**	25,965 ± 7,010 (6)^(b)^
*Insr*	18,149 ± 2,080 (4)	17,441 ± 1,248 (4)	13,880 ± 477 (4)^(a)^
*Irs1*	60,068 ± 12,861 (6)	36,282 ± 2,983 (6)	11,888 ± 2,316 (6)**^(b)^
*Irs2*	88,190 ± 14,430 (6)	81,351 ± 6,149 (6)	24,811 ± 3,661 (6)**^(b)^
*Pgc1a*	1,776 ± 441 (6)	1,009 ± 87 (6)	1,070 ± 388 (6)
*Pgc1b*	7,558 ± 1,261 (5)	6,282 ± 737 (4)	4,042 ± 299 (5)^*(a)^
Skeletal muscle			
*Ccl2*	204 ± 46 (6)	554 ± 131 (6)*	1,224 ± 111 (6)**^(b)^
*Cxcl1*	89 ± 9 (6)	70 ± 13 (6)	129 ± 15 (6)^*(a)^
*Cd68*	3,927 ± 643 (6)	4,237 ± 445 (6)	10,178 ± 422 (6)**^(b)^
*Cd11b*	2,239 ± 361 (6)	3,463 ± 521 (6)	3,956 ± 773 (6)
*Emr1*	387 ± 25 (6)	421 ± 57 (6)	724 ± 41 (6)**^(b)^
*Adipoq*	94,837 ± 17,077 (6)	116,610 ± 56,998 (6)	114,194 ± 19,422 (6)
*Glut4*	512,548 ± 27,756 (6)	379,390 ± 30,641 (6) **	423,423 ± 22,047 (6)*
*Insr*	57,791 ± 2,283 (6)	42,298 ± 1,673 (6) **	50,984 ± 2,188 (6)^(a)^
*Irs1*	58,141 ± 7,790 (6)	45,326 ± 6,154 (6)	47,738 ± 5,014 (6)
*Irs2*	51,152 ± 9,399 (6)	27,292 ± 4,061 (6)*	21,470 ± 1,374 (6)*
*Pgc1a*	39,951 ± 24,961 (6)	17,510 ± 8,171 (6)	12,085 ± 1,107 (6)
*Pgc1b*	6,003 ± 1,476 (6)	7,408 ± 1,327 (6)	10,150 ± 271 (6)*

## ABBREVIATIONS

A-SAA: Acute-phase serum amyloid A
CCL2: Chemokine (C-C motif) ligand 2
CXCL1: Chemokine (C-X-C motif) ligand 1
DIO: Diet induced obesity
HFD: High-fat diet
IBMX: 3-isobutyl 3-methylxanthine
PGC: Peroxisome proliferator activated receptor-*γ* coactivator
PPAR: Peroxisome proliferator activated receptor
SAA: Serum amyloid A
TBP: TATA box binding protein
1 w HFD: One week of HFD
16 w HFD: 16 weeks of HFD
